# Actions of Retinoic Acid in the Pathophysiology of HIV Infection

**DOI:** 10.3390/nu14081611

**Published:** 2022-04-12

**Authors:** Neil Sidell, Maureen A. Kane

**Affiliations:** 1 Department of Obstetrics and Gynecology, Emory University School of Medicine, Atlanta, GA 30322, USA; 2 Department of Pharmaceutical Sciences, University of Maryland School of Pharmacy, Baltimore, MD 21201, USA

**Keywords:** retinoic acid, HIV, mucosal immunity, retinoids, immune regulation

## Abstract

The vitamin A metabolite all-trans retinoic acid (RA) plays a key role in tissue homeostasis and mucosal immunity. RA is produced by gut-associated dendritic cells, which are among the first cells encountered by HIV. Acute HIV infection results in rapid reduction of RA levels and dysregulation of immune cell populations whose identities and function are largely controlled by RA. Here, we discuss the potential link between the roles played by RA in shaping intestinal immune responses and the manifestations and pathogenesis of HIV-associated enteropathy and similar conditions observed in SIV-infected non-human primate models. We also present data demonstrating the ability of RA to enhance the activation of replication-competent viral reservoirs from subjects on suppressive anti-retroviral therapy. The data suggest that retinoid supplementation may be a useful adjuvant for countering the pathologic condition of the gastro-intestinal tract associated with HIV infection and as part of a strategy for reactivating viral reservoirs as a means of depleting latent viral infection.

## 1. Retinoids in Gut Physiology

**Figure 1:**
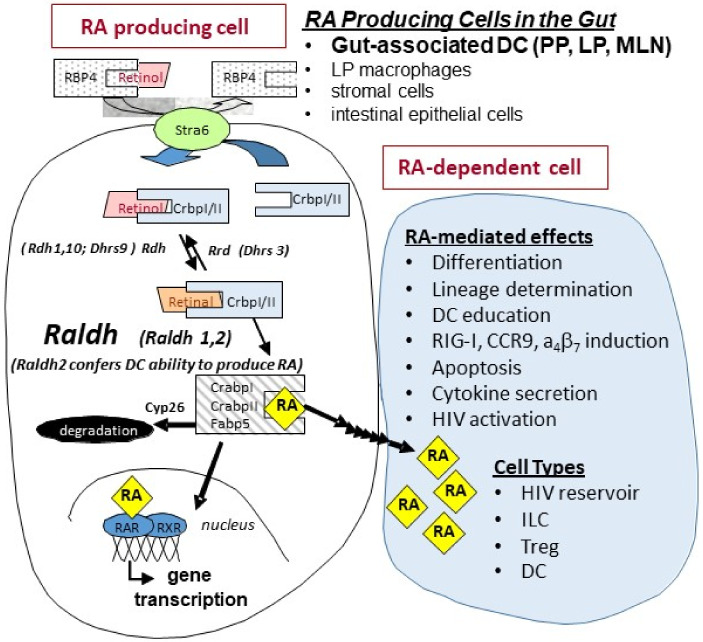
**Retinoid metabolism and RA signaling**. Studies in mice and humans have indicated that major cellular sources of all-trans retinoic acid (RA) production in the intestine are dendritic cells (DCs) from mesenteric lymph nodes (MLN), Peyer’s patches (PP), and small intestine lamina propria (LP); intestinal epithelial cells; and macrophages [1,2,12]. Raldh2 expression is restricted to limited cell types and is the critical regulatory element in immune cell populations that confers cells the ability to synthesize RA. Once formed, RA can be: (1) transported to the nucleus of the RA-producing cell (e.g., dendritic cells in the gut) where it binds to nuclear receptors and initiates gene transcription, (2) transported to neighboring cells (e.g., T-cells and ILCs) to initiate RA-mediated signaling, and/or (3) degraded by Cyp26.

Vitamin A is an essential nutrient, which enacts its biological functions through its active metabolite, all-trans retinoic acid (RA). RA plays a vital role in the function of mucosal tissue as well as innate and adaptive immunity [1,2,3,4,5,6,7,8,9,10]. For example, RA is essential to intestinal epithelial cell differentiation and barrier function [1,3,11], imprinting the innate immune system [1,3,12], inducing gut-homing receptors CCR9 and α4β7 on T cells, regulating innate lymphoid cells (ILCs) [4,13,14], and inducing gut tropism in B cells [15,16]. The ability of immune-relevant cells to metabolize vitamin A and produce RA [1,2,17,18,19] is regulated through the activity of retinal dehydrogenase (Raldh) enzymes that control the oxidation of retinal to RA (Figure 1).

Retinoid metabolism and the abundance of active metabolite RA are regulated by a series of binding proteins and enzymes in a cell- and context-specific manner. Generally, in cells that produce RA, cellular retinol-binding protein, type 1 (CrbpI) delivers retinoids to specific enzymes to facilitate the production of RA, including delivery of retinol-to-retinol dehydrogenases (Rdh) for conversion into retinal; retinal-to-retinal reductases (Rrd) for conversion into retinol; and retinal-to-retinal dehydrogenases (Raldh) for conversion into RA. The intestine is unique in that it is also the major site of vitamin A absorption; specifically, enterocytes are the major type of intestinal epithelial cell important to nutrient absorption. Enterocytes express high levels of CrbpII, which directs absorbed dietary-derived retinoids away from RA production to protect against excessive RA levels that would cause retinoid toxicity, including delivery of retinal to Rrd for reduction into retinol and delivery of retinol to lecithin:retinol acyltransferase (Lrat) for esterification into retinyl esters, which are incorporated into chylomicrons for systemic transport [20].

A primary source of Raldh-expressing cells that represent sources of RA in the intestinal mucosa are myeloid dendritic cells (DCs) that reside in the Peyer’s patch, small intestine lamina propria (LP), and mesenteric lymph node [4,14,21]. Production of RA by DCs is crucial to the differentiation of T cells to Foxp3+ Treg [17,22,23,24]. RA enhances ILC plasticity and induces the production of IL-22 in group 3 ILC cells (ILC3) [25,26,27], which, among other actions, helps regulate the integrity of the intestinal epithelial cells (IEC). Additionally, RA has been shown to attenuate gut inflammation [25,26,28,29,30] where its local production in the gut is essential for maintaining proper gut homeostasis and innate immune responses [17,31,32,33]. Mucosal damage in the small intestine induces a local state of vitamin A deficiency (VAD), where limited RA availability inhibits the number and function of cell populations important to gut homeostasis and immune response that rely on the RA signal [4,14,15,23,31,32,34,35]. To this end, gut-associated DCs and immune cells from patients with inflammatory bowel diseases (IBD, e.g., Crohn’s disease and ulcerative colitis) express lower Raldh and have impaired functions that are dependent upon RA [36]. Extensive literature in rodent models of IBD has shown that RA treatments can significantly attenuate established IBD disease by increasing the number and function of CD103+ DCs and Tregs while reducing Th17 cells [23,37,38,39,40,41,42,43,44,45,46].

## 2. Mucosal Damage Results in RA Deficiency and Depletion of RA-Dependent Cell Populations

**Figure 2:**
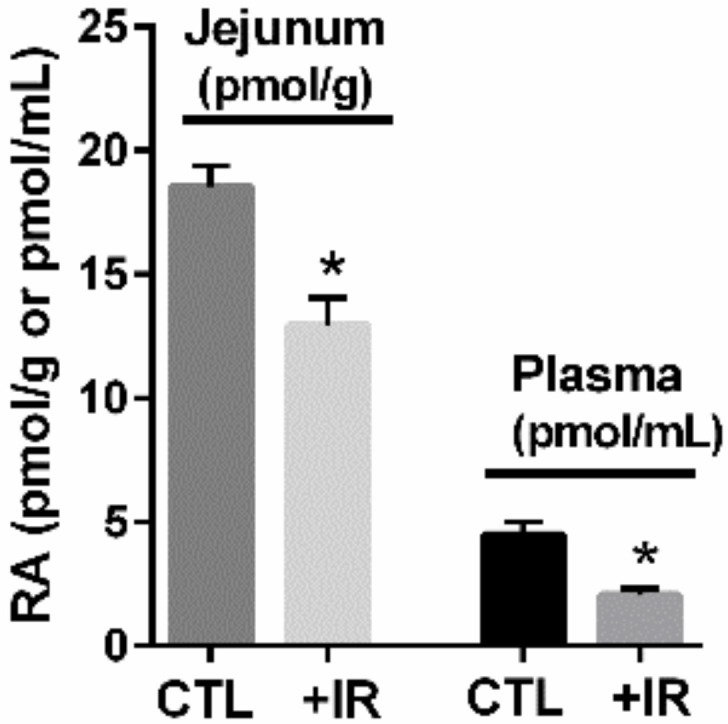
**GI mucosal damage results in loss of small intestine RA, which is reflected in reduced plasma RA levels**. Loss of gut RA (jejunum, left) resulted in reduced plasma RA levels (right) in a partial body irradiation (PBI) with 5% bone marrow sparing (BM5) non-human primate (NHP) model of radiation-induced gut damage (rhesus macaques; 11.5 Gy PBI/BM5, *n* = 10; control, *n* = 10) as quantified by liquid chromatography-tandem mass spectrometry (LC-MS/MS); mean ± SEM (* *p* < 0.05). Adapted from Yu J, Huang W et al., 2021 [47].

Mucosal damage results in a local state of functional vitamin A deficiency with reduced levels of active metabolite RA and a selective depletion of RA-dependent DC subsets in the small intestine [32]. We observed a similar local vitamin A deficiency in non-human primates (NHP) with reduced RA in the small intestine (jejunum) after mucosal injury, which was accompanied by reduced plasma RA (Figure 2 and [32,35,47]). As small intestine is a region of high RA production and is a major contributor to plasma RA levels, recent studies in NHP show a strict correlation between RA concentrations in intestinal mucosa following radiation-induced mucosal damage and circulating RA plasma levels (Figure 2 and [32,35,47]). In this regard, NHP models of radiation-induced mucosal injury are used to model immune dysfunction in bone marrow transplant patients and display similar histological damage to HIV/SIV infection, including loss of epithelial integrity, inflammation, villus blunting, and loss of select cell populations. Both radiation-induced mucosal injury and diet-induced vitamin A deficiency resulted in a similar decrease in the number of DC in the small intestine LP. Treatment of the diet-induced vitamin A deficiency with RA restored the number of DC to a level similar as control, whereas treatment of control animals with an RA-blocking agent resulted in a decrease in the number of small intestine LP DCs similar to diet-induced vitamin A deficiency and radiation-induced mucosal injury [32]. This study showed that select RA-dependent cell populations can be modulated by blocking or potentiating the action of RA in the small intestine [32]. It also showed that after mucosal injury, RA provided a differentiation prompt that controlled the fate commitment of opposing pre-DC derived lineages in gut, demonstrating that this single RA nutrient can critically impact cell-mediated immunity and gut cell homeostasis [32].

## 3. HIV and Gut Immunity

A burgeoning body of evidence suggests that dysregulation of innate immunity in the gut mucosae plays an important role in HIV/SIV pathogenesis and invites the hypothesis that defective RA signaling is implicated in the early gut pathology of HIV infection. To this end, NK cells and ILC3 are two innate effector cells that are regulated by RA in their development and trafficking programs for maintaining proper gut homeostasis [48]. NK cells expand in blood during the acute HIV infection period [49,50] prior to the development of CD8+ T lymphocytes and can mediate immune selection pressure in HIV-infected individuals [50]. Moreover, long-term non-progressors have increased NK cell cytotoxicity compared to viremic individuals [51]. NK cells can also inhibit CCR5-dependent entry of HIV by secreting β-chemokines CCL3, CCL4, and CCL5 [52]. In rhesus macaques, NK cells have been shown to lyse SIV-infected cells [53] and cells pulsed in vitro with the simian immunodeficiency virus SIV_mac_ [54]. Acute infection of rhesus macaques with SIVmac251 induces rapid NK cell activation and increased cytotoxicity [55], and longitudinal studies suggest that NK cells may be associated with preventing disease progression [56,57,58,59]. A major correlate of NK cell-mediated activity in control of virus replication is reduced NK cell terminal differentiation and exhaustion, in lieu of NK repertoire diversity [60]. Studies in NHP have suggested a role for RA in this activity [61]. To this end, experimental therapies that can significantly reduce the frequency of terminally differentiated NK cells were shown to coincide with increased RA levels [61]. Similarly, ILC3 cells produce IL-22 and/or IL-17, but depend on the retinoid orphan receptor type γt (RORγt) for development [62,63,64,65,66,67]. RORγt expression requires upstream RA induction of retinoic acid receptor (RAR) activity that controls the RORγt locus [68]. ILC3 from rhesus macaques were found to be enriched in oral, Gl tract, and genital mucosal tissues, express high levels of RORγt, and produce IL-17 and IL-22 [34,69,70,71,72,73,74], making them analogous to human ILC3. ILC3-produced IL-22 and IL-17 regulate integrity of the gut epithelium, mediated in part by the ability of IL-22 to stimulate gut epithelial cells, resulting in upregulation of mitogenic and anti-apoptotic molecules [64,75,76,77,78]. It has been shown that ILC3 populations are massively depleted in the gastrointestinal tract (GIT) and IL-17 production is suppressed during SIV infection by increased immune activation [34,69,70,71,72,73], but can be restored following treatments that are associated with increased RA levels [61].

**Figure 3:**
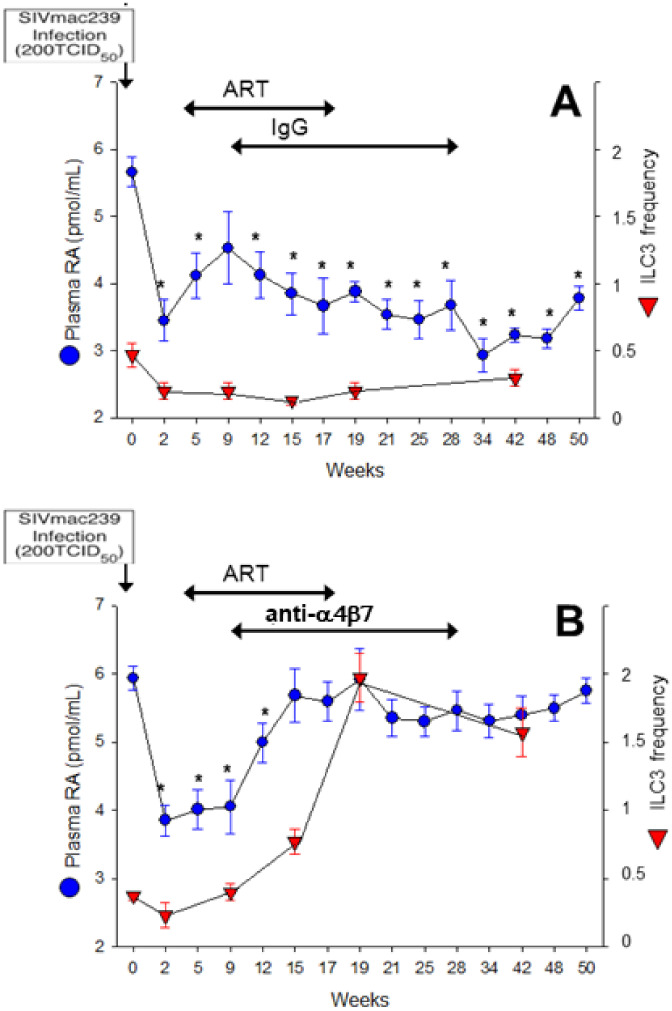
**Effects of acute SIV infection and anti-α4β7 antibody treatment on RA production and ILC3 frequency in NHP**. NHP (Rhesus macaques) were infected with SIV at week 0 and treated with daily anti-retroviral therapy (ART) and control IgG (*n* = 7) (**A**) or ART and anti-α4β7 antibody (*n* = 8) (**B**) over the post-infection treatment periods indicated by the black arrows. (**A**) The ART/IgG regimen showed a rapid decrease in RA levels (blue) along with a decrease in the mean frequencies of ILC3 cells (red). (**B**) The ART/anti-α4β7 antibody regimen resulted in a recovery of plasma RA levels by week 15 (blue) equivalent to the pre-infection values that was accompanied by an expansion of the ILC3 cells (red). The ILC3 were obtained from the gated population of CD3−/CD8+/HLA-DR+/NKG2a−/NKp44+ cells in samples of colorectal tissue-isolated mononuclear cells from 3 representative animals from each group. RA levels (mean ± SEM) were quantified by LC-MS/MS and compared with pre-infection (week 0) values (* *p* < 0.05). Adapted from Byrareddy SN et al., 2016 [61].

**Figure 4:**
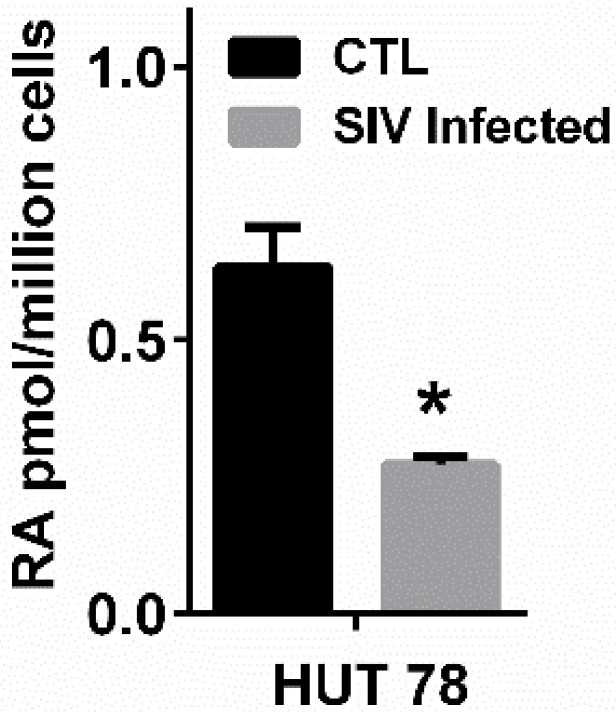
**Effects of SIV Infection on RA production in CD4+ T cells**. Hut78 cells (CD4+ T cells) or chronically SIV-infected Hut78 cells [86] were treated with 2 µM retinol (substrate for RA synthesis) under serum free conditions for 4 h [87]. RA levels in cell pellets were quantified by LC-MS/MS [88]. SIV infection reduced the ability of Hut78 cells to make RA. * *p* < 0.05.

**Figure 5:**
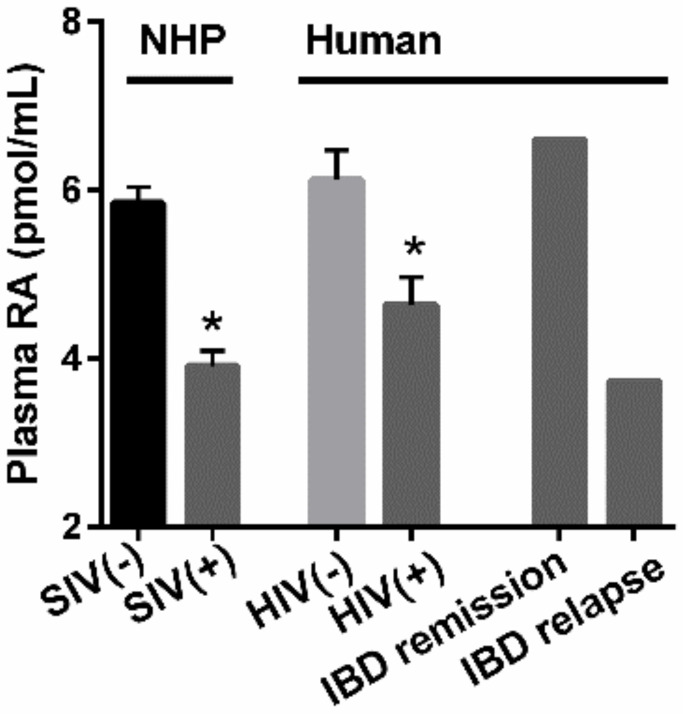
**SIV-infected NHP and HIV-infected humans have similar decreases in plasma RA levels, which correlate with decreases in plasma RA levels observed in IBD relapse as compared to IBD remission**. NHP (SIV+/−) and human (HIV+/−) plasma RA levels were quantified via LC-MS/MS, mean ± SEM. SIV (−), *n* = 10; SIV (+) 50 weeks, *n* = 5; HIV (−), *n* = 6; HIV (+) for ≥52 weeks, *n* = 6. IBD remission and IBD relapse levels were median plasma RA levels observed in *n* = 38 and *n* = 24 patients, respectively from a study by Paul et al. 2018 [85] (* *p* < 0.05). Adapted from Byrareddy SN et al., 2016 [61].

As RA-producing mucosal DCs are among the first cells that encounter HIV/SIV, we postulated that acute infections may be reflected by rapid reduction in RA levels. In an NHP model of SIV, an acute state of VAD was observed after SIV infection, as reflected by reduced plasma RA (Figure 3A). Plasma RA continued to decline with disease progression. These plasma retinoid measurements revealed that substrates for RA production (retinol) and stored retinoids (retinyl esters) were not impacted by SIV infection, indicating that reductions in RA were due to a defect in metabolism to produce RA [61]. Preliminary in vitro studies using a human CD4+ T-cell line supported the observations that SIV infection directly inhibits RA production (Figure 4) while measurement of plasma RA in HIV-infected humans shows reduced levels of RA similar to those of SIV-infected NHP (Figure 5). We have also observed a decrease in duodenal RA levels in HIV-infected patients consistent with the extent of depletion reflected by plasma RA (unpublished data). ILC3 were also reduced after acute SIV infection, a reduction that persisted over time (Figure 3A). Anti-retroviral therapy (ART) did not correct either the deficit in RA or the reduced number of ILC3. Experimental treatment with a primatized anti-α4β7 antibody resulted in a recovery of plasma RA levels equivalent to uninfected NHP and was accompanied by an expansion of ILC3 (Figure 3B), CD4+ T-cells and non-terminally differentiated NK cells in the GIT, conditions which are associated with improved gut homeostasis [61]. Although the effects of this experimental treatment on viral load are, controversial [79,80,81], its effects on immune parameters and RA levels have not been in dispute. In a recent study by Frank et al. [82], treatment of simian-human immunodeficiency virus-infected rhesus macaques with anti-α4β7 was shown to affect their immune response in such a way as to prolong virologic control induced by anti-HIV broadly neutralizing antibodies. An unbiased analysis of immune and gastrointestinal parameters responding to anti-α4β7 therapy in SIV-infected NHP revealed that RA was the first correlate of response related to gut homeostasis, closely followed by cell populations whose identities are largely controlled by RA [61]. To this end, RA and RA-modulated NK cells and ILC significantly expanded with the initiation of therapy. This coupling between treatment and ILC number is consistent with findings in IBD patients that effective treatment with anti-α4β7 antibody (vedolizumab-VDZ) is closely associated with changes in innate rather than adaptive immunity [83]. Additionally, in the first reported human study in HIV-infected patients with IBD [84], VDZ therapy reduced the size and number of lymphoid aggregates in the terminal ileum and also increased NK cell frequency and activation after VDZ treatment [84]. In parallel to this observation, a human study of IBD patients treated with VDZ showed that RA levels assayed post-VDZ treatment were correlated with improvements in gut homeostasis [85]. IBD patients that were in remission maintained higher RA levels, similar to healthy controls, while those that experienced relapse had reduced RA levels, similar to those seen under HIV/SIV-infected conditions [85] (Figure 5). The mechanisms that contribute to the re-establishment of mucosal homeostasis during VDZ treatment in HIV patients are not fully understood.

## 4. Therapeutic Potential of RA to Modify HIV Pathology

**Figure 6:**
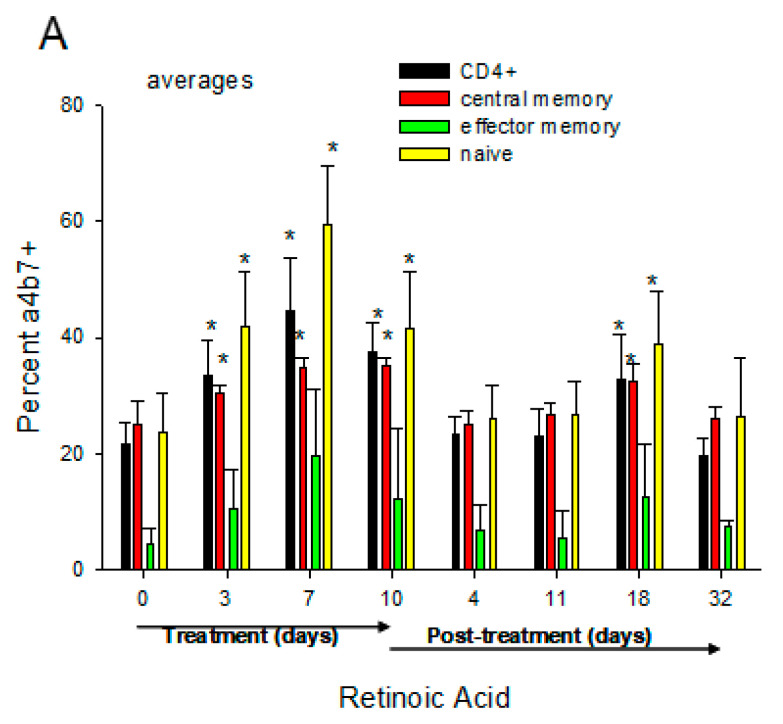

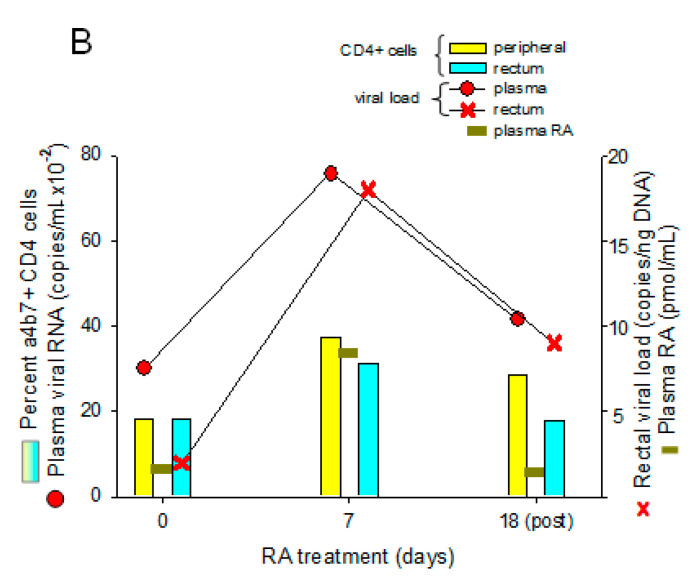
**In vivo administration of RA to macaques with low viral loads**. Monkeys were fed 10 mg/kg/day all-trans RA (orally) for 10 days, then followed for another 32 days. (**A**) Increased α_4_β_7_ expression on CD4+ lymphocytes. Central memory, effector memory, and naïve CD4+ lymphocytes were gated based on their dual expression of CD28 and CD95 [4] and quantified for percent α_4_β_7_+ cells. Naïve CD4^+^ T cells were categorized as CD28^+^/CD95^−^, central memory CD4^+^ T cells as CD28^+^/CD95^+^, and effector memory CD4^+^ T cells as CD28^−^/CD95^+^. Results represent the mean ± SEM of 4 monkeys. * *p* < 0.05, significant difference compared with baseline (day 0) values. (**B**) Representative example of a “spontaneous controller” monkey from “A” showing the correlation of plasma and rectal viral load with RA levels and expression of α_4_β_7_. Adapted from Olwenyi OA, 2020 [93].

**Figure 7:**
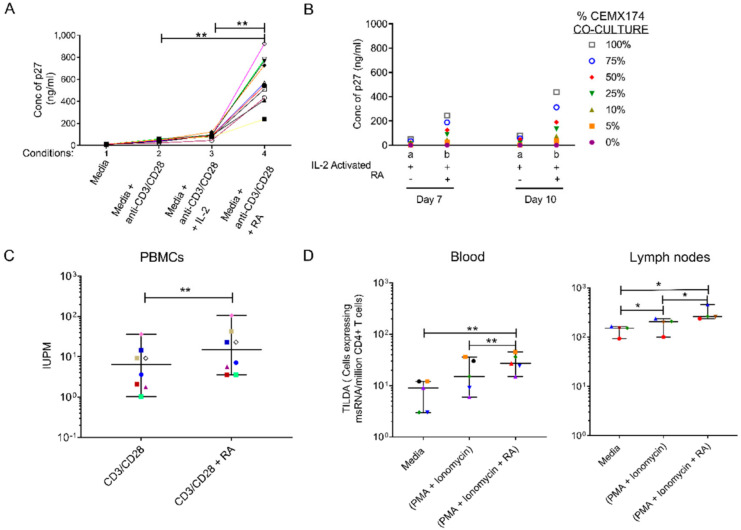
**Retinoic Acid (RA) enhances viral replication in vitro and improves detection of the viral reservoir**. (**A**) Levels of p27, a regulatory protein produced by HIV/SIV-infected cells [94], were measured in supernatant of peripheral blood mononuclear cells (PBMCs) from 10 randomly selected naive rhesus macaques (RMs). The PBMCs were infected with SIVmac239 after in vitro culturing with (1) media, unstimulated (2) anti-CD3/CD28 beads to activate the T-cell receptor (3) anti-CD3/CD28 + IL-2 (10 U/mL) and (4) anti-CD3/CD28 beads + IL-2 together with RA (1 μM) added under separate conditions on day 10. (**B**) Levels of p27 detected in supernatant fluids from pooled (*n* = 10 RMs) enriched CD4 T cells that were treated with either (a) anti-CD3/CD28 and IL-2 only or (b) anti-CD3/CD28 + IL-2 and RA. Thereafter, viral expansion was carried out by the co-culture with CEMX174 cells across different concentrations (0 to 100%) on days 7 and 10 respectively. (**C**) Quantitative Viral Outgrowth Assay (QVOA) of CD4^+^ T cells purified from PBMCs of anti-retroviral therapy (ART)-suppressed macaques indicating levels of Infectious Units per Million (IUPM) in CD3/CD28 versus CD3/CD28 + RA conditions (*n* = 8). (**D**) levels of msRNA transcripts obtained from enriched CD4 T cells collected from PBMC (*n* = 5 RMs) or auxiliary lymph node CD4^+^ T cells (*n* = 4 RMs) that were cultured in media only, media plus phorbol myristate acetate (PMA) + ionomycin and media plus PMA + ionomycin supplemented with retinoic acid. * shows *p* < 0.05 and ** represents *p* < 0.001 significant difference across studied groups obtained using Wilcoxon matched pairs signed rank tests. Reproduced with permission from Figure 1 from Olwenyi OA et al. [93].

We have demonstrated that oral daily administration of RA to chronically SIV-infected monkeys with low viral loads (so-called “spontaneous controllers”) induced an increase in α4β7+ CD4 T cells, most apparent in the “naïve” subpopulation, and led to moderate increases in plasma and gut (rectal) viral loads (Figure 6). The mechanism of this latter effect is unknown, but may be related to the ability of RA to upregulate α4β7 since HIV gp120 has been shown to bind and signal through α4β7, which can trigger cellular activation [89] and increased viral production. Several studies have shown that the in vitro stimulation of CD4+ T cells with RA enhances surface expression of α4β7 [90,91]. Thus, the possibility that RA can re-activate viral reservoirs should be considered as a potential mechanism of action. To this end, reactivation of latent HIV/SIV by AMPK-activating agents including RA has been demonstrated [92]. This hypothesis is supported by recent studies that confirmed the ability of RA to enhance the re-activation of replication-competent viral reservoirs in peripheral rhesus macaque CD4+ T cells following activation with anti-CD3/CD28 beads to activate the T-cell receptor or PMA/ionomycin [93] (Figure 7).

Likewise, Zhang et al. showed that supplementation of RA during assays designed to estimate viral reservoirs showed an increase in viral replication in RA-treated versus without RA conditions [95]. These studies are supported by the findings of Li et al. [96] who showed the ability of acitretin, an FDA-approved homologue of RA, to reactivate latent HIV transcription and promote apoptosis of CD4+ T cells from HIV patients on suppressive ART. This latter effect was shown to be mediated at least in part through a retinoic acid-inducible gene I (RIG-I)-mediated innate response [96]. In contrast, studies by Garcia-Vidal et al. could not reproduce these findings of HIV reactivation or induced apoptosis by acitretin, although its ability to upregulate RIG-I was confirmed [97]. These conflicting results may have root in the unique mechanism by which acitretin acts on the retinoid pathway; instead of directly binding to the RA nuclear receptors (RARs), acitretin displaces RA from CRABPI, a binding protein that channels RA to CYP26 for degradation, (see Figure 1) effectively raising free cellular RA levels to increase RAR receptor occupancy [98,99,100]. Thus, differences in culture conditions that can affect the intrinsic intracellular production of RA (e.g., retinol levels in the medium), or the inherent ability of certain cell types to synthesize RA may have profound effects on the activity of acitretin. With regard to the latter, it should be noted that the acitretin studies cited above utilized cultures of isolated T-cells which are inherently poor RA producers in comparison to many other cell types in the GIT [2,9,21]. Taken together, the findings suggest that RA may be useful as part of a “shock and kill” strategy through its ability to enhance viral reservoir activation [92].

The first interventional clinical trial to examine the direct in vivo effects of RA supplementation using isotretinoin (13-cis-RA) in HIV patients on anti-retroviral therapy (ART) has recently been completed [101]. This first-in human study characterized the effects of isotretinoin treatment on systemic immune activation, HIV latent reservoir levels, and CD4+ T cell reconstitution in the blood. The study results suggest that isotretinoin treatment, in combination with ART, may reduce the HIV reservoir, as measured by HIV cell associated-DNA, while providing immunologic benefits, such as an increase in peripheral CD4+ T cell counts. Treatment with isotretinoin also increased plasma all-trans RA, which may be important to the observed effects. Larger clinical trials will be needed to further evaluate the mechanism and effectiveness of isotretinoin as an adjuvant treatment for people living with HIV.

## 5. Conclusions

**Figure 8:**
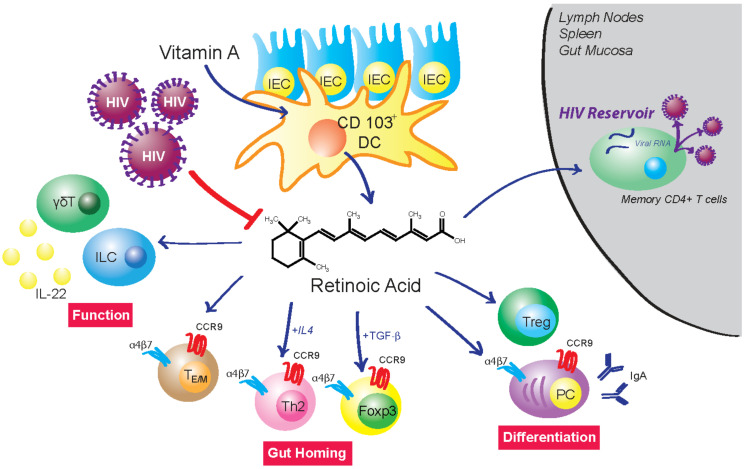
**HIV infection disrupts RA-mediated control of intestinal homeostasis**. Mucosal CD103^+^ dendritic cells (DC) express Raldh2 and are a major source of RA production in the gut. They are among the first cells encountered by HIV and their infection inhibits RA biosynthesis. Studies have shown that acute HIV/SIV infection is closely linked to disruption of cell populations whose identity and function are regulated by RA. RA controls the differentiation of T cells into Tregs and B cells into IgA-producing plasma cells (PC). It also regulates the migration of lymphoid cells into the intestine by induction of gut-homing receptors α_4_β_7_ and CCR9. RA enhances IL-22 production from γδT cells and innate lymphoid cells (ILC). IL-22 plays a vital role in the regulation of multiple aspects of gut epithelial integrity, including control of epithelial cell growth and permeability, production of mucus and antimicrobial proteins, and complement production. RA supplementation can enhance activation of replication-competent HIV reservoirs. These data suggest the possible therapeutic use of retinoid derivatives to counteract HIV-associated enteropathy and as part of a “shock and kill” strategy to deplete latent viral infection.

In the GIT, RA plays a critical role in tissue homeostasis and mucosal immunity through regulation of a variety of immune cell functions, including proinflammatory/suppressor T-cell balance, ILCs, generation of IgA-antibody secreting cells, and maturation/differentiation of myeloid lineage cells (Figure 8). A primary source of Raldh-expressing cells that represent sources of RA in the GIT is CD103+ DCs. These gut-associated DCs from patients with IBDs express lower Raldh and have impaired functions that are dependent upon RA. During acute HIV-1 infection, there is a marked depletion of RA associated with major changes in the distribution of immune cell subsets that we submit are the basis for the initiation of GIT damage and the facilitation of the seeding of viral reservoirs. Since mucosal DCs are among the first cells that encounter HIV/SIV, it is not surprising that acute infection is reflected by rapid reduction of RA levels. The mechanism of the reduced RA production resulting from HIV infection has yet to be determined. This action is followed by impaired immune functions that are dependent on physiological levels of RA. Thus, it seems logical that attempts to modulate these changes with the use of RA during this acute infection period may have profound effects on the subsequent course of infection and disease progression. During chronic HIV infection, there is a continued decrease in the production of RA that is associated with disease progression and suppression of immune cell functions that are, in part, dependent on RA. The decrease in RA production and immune cell dysfunction is not totally reversed by the administration of ART and thus suggests that adjunct immune therapies, including the use of RA, should be considered in efforts to reverse this disease course. Finally, the demonstrations that RA can enhance activation of replication-competent HIV/SIV reservoirs suggest the interesting possibility that exogenous administration of RA may be useful as part of a strategy to reactivate viral reservoirs as a means of depleting latent viral infection.

## Data Availability

Not applicable.
